# Editorial: Research and advances on medication for corneal diseases and surgery

**DOI:** 10.3389/fphar.2024.1434180

**Published:** 2024-07-12

**Authors:** Nan-Ji Lu, Farhad Hafezi, Carina Koppen, Ioannis M. Aslanides

**Affiliations:** ^1^ Department of Ophthalmology, West China Hospital, Sichuan University, Chengdu, China; ^2^ ELZA Institute, Dietikon, Switzerland; ^3^ Department of Ophthalmology, Antwerp University Hospital, Edegem, Belgium; ^4^ Emmetropia Mediterranean Eye Institute, Heraklion, Greece

**Keywords:** cornea, medication, pharmacology, corneal cross linking, PACK-CXL, corneal neovascularization, corneal endothelial disease

The aim of this Research Topic, “Research and Advances on Medication for Corneal Diseases and Surgery,” is to gather the latest developments from researchers and clinicians working on medication for challenging corneal diseases and surgeries. The guest editorial team extends its gratitude to all colleagues who submitted their reviews and research articles for this Research Topic.

As an emerging and cutting-edge technology, corneal cross-linking (CXL) is a photochemical interaction reaction, which originally uses a chromophore—riboflavin and 365 nm ultraviolet A (UVA) light to generate oxygen free radicals and activate various pathways. These effects create covalent bonds in the cornea to improve corneal biomechanics for the treatment of corneal ectasias (Wollensak et al., 2003). Later, as comprehensively reviewed by Qin et al., other chromophores (crosslinking agents), and their enhancers and supplements were developed to improve the effectiveness of CXL while maintaining corneal safety.

In addition to enhancing corneal biomechanical properties, subsequent research identified the antimicrobial function of CXL, leading to the term “photoactivated chromophore for keratitis-corneal cross-linking (PACK-CXL)” for this use (Hafezi and Randleman, 2014). Olshaker et al. evaluated the efficacy and safety of high fluence PACK-CXL (30 mW/cm^2^, 5 min 33 s, 10.0 J/cm^2^) at the slit lamp for patients with bacterial, fungal, or mixed-origin keratitis as an adjunct therapy to conventional antimicrobial therapy. They found that this PACK-CXL protocol was safe with no complications observed. While PACK-CXL has several mechanisms of antimicrobial action, including generating reactive oxygen species to directly kill pathogens (Kling et al., 2020; Lu et al., 2023a; Lu et al., 2023b) and rendering the cornea more resistant to collagenase digestion (Aydemir et al., 2024; Hafezi et al., 2024), the reduced corneal transmission after infection (Lu *et al.* manuscript in preparation) inspires us to continue optimizing the PACK-CXL protocols for timely administration on the slit lamp to improve the prognosis of infectious keratitis (Hafezi et al., 2021; Hafezi et al., 2022).

Corneal neovascularization (CNV) is a serious disease worldwide that damages corneal transparency, which can result in impairing vision and cause blindness. The treatments for CNV include surgery and medication, and the latter one is currently the main approach. Shi et al. developed the optimal formulation of topical Sunitinib microemulsion (STB-ME) eye drops to inhibit CNV. Their animal study showed that this new medication has similar efficacy to dexamethasone, with sustained release and high ocular bioavailability.

Corneal endothelial disease is a global sight-threatening disease, in addition to Fuchs’ endothelial dystrophy, the most common cause of endothelial decompensation is phacoemulsification during the cataract surgery. Jin et al. found that the addition of Ala-Gln in irrigation solutions may inhibit the destruction of the corneal endothelial barrier function and pump function caused by acute ocular hypertension. This finding may offer a new approach to preventing damage to endothelial cells during surgery.

In summary, this Research Topic has gathered several aspects of developing new techniques and topical medications for various corneal diseases. More specifically, it illustrates the promising effects of utilizing CXL in treating corneal ectasias and infectious keratitis, as well as developing new topical medications for treating CNV and preventing corneal endothelial disease. The development of corneal topical medications combining pharmacology and biomedical engineering holds great promise, and the current Research Topic is expected to provide a valuable reference for exploring this research field.
